# How General Practitioners and Their Patients Adhere to Osteoporosis Management: A Follow-Up Survey among Czech General Practitioners

**DOI:** 10.3389/fphar.2017.00258

**Published:** 2017-05-11

**Authors:** Magda Vytrisalova, Tereza Touskova, Leos Fuksa, Roman Karascak, Vladimir Palicka, Svatopluk Byma, Jan Stepan

**Affiliations:** ^1^Department of Social and Clinical Pharmacy, Faculty of Pharmacy in Hradec Kralove, Charles UniversityHradec Kralove, Czechia; ^2^Faculty of Medicine in Hradec Kralove, Charles University, Hradec Kralove and University Hospital Hradec KraloveHradec Kralove, Czechia; ^3^Department of Social Medicine, Faculty of Medicine in Hradec Kralove, Charles UniversityHradec Kralove, Czechia; ^4^Department of Rheumatology, Faculty of Medicine, Institute of Rheumatology, Charles UniversityPrague, Czechia

**Keywords:** general practitioners, osteoporosis, adherence to management, patient adherence, calcium intake, knowledge, treatment guidelines, barriers

## Abstract

**Introduction:** General practitioners (GPs) are key participants in osteoporosis (OP) management. The aim was to evaluate their adherence to lege artis management of the disease, potential barriers, and to discuss differences observed in comparison with the baseline survey carried out in 2007; the focus was on secondary prevention.

**Methods:** On behalf of two professional associations, 2-round postal survey among randomly selected GPs (>1/4 of all Czech GPs) was performed in 2014. The questionnaire covered areas concerning GP's role in the fight against OP, knowledge about OP, management of OP-related fractures, barriers to the management of OP, system- and patient-related in particular, and availability and use of information sources.

**Results:** The overall questionnaire return rate was 37% (551 respondents); mean age of the respondents was 53 year (37% men). The GP's role in the treatment of OP was rated as essential in 28 and 37% of men and women, respectively (*P* = 0.012). The guideline for diagnosis and treatment of OP for GPs was considered accessible by 92% of respondents. As much as 60% of the respondents were adherent to the guideline, i.e., used it repeatedly. The knowledge of several risk factors was very good, however, recommended daily intake of calcium was stated correctly by only 41% of respondents, and daily intake of vitamin D by only 40%. Three quarters reported active steps after a fracture: referral to a specialist, life-style recommendations, prescription of calcium/vitamin D supplements. Half of the respondents focus on fall prevention. System-related barriers, such as lack of possibility to prescribe selected drugs (61%) and financial limits set by health insurance company (44%) were most frequently reported. Patient-related barriers were also common, patient's non-adherence (reported by 29%) and patient's reluctance to go to a specialist (18%).

**Conclusion:** GPs adhered to OP management more than in 2007. Knowledge of risk factors and involvement in post-fracture care was relatively high. Compared to baseline survey, patient-related barriers, patient non-adherence in particular, were more common. Prescribing conditions are still an important issue. Among GPs, education should be focused on calcium and vitamin D intake, doses, sources, and supplements.

## Introduction

Osteoporosis (OP) is a systemic disease characterized by low bone mass and deterioration of bone tissue, with a consequent increase in bone fragility and susceptibility to fracture (Kanis et al., 2013). Fractures of proximal femur and vertebral fractures are the most serious types, both clinically and economically, associated with decreased quality of life, disability, loss of independence, and even mortality. As many as 22 million women and 5.5 million men suffered from OP in the EU in 2010. The costs of osteoporosis-related fractures were estimated 37 billion EUR. Incident fractures represented 66% of this burden, long-term post-fracture care 29%, and medications 5% (Svedbom et al., 2013).

In the Czech Republic, approximately 72,000 new fragility fractures were sustained in 2010. When adjusting the demographic projections for 2025, the number of incident fractures was estimated at 94,000 in 2025, representing an increase of 21,000 fractures (Svedbom et al., 2013). Approximately 9,300 women and 3,500 men aged over 50 years suffer fractures of the proximal femur every year (Štěpán et al., 2015). The remaining lifetime probability of hip fracture at the ages of 50 years in men and women was estimated to approximately 7 and 15%, respectively (Stepan et al., 2012).

Pharmacological treatment significantly decreases fracture risk. All patients being considered for treatment of OP should be also counseled on risk factor reduction including the importance of calcium and vitamin D intake, and physical activity as part of any treatment program for OP. Despite advances in diagnosis, treatment and management of OP at population level, large number of people with OP and/or osteoporosis-related fracture still escape attention. It is estimated, the proportion of persons over the age of 50 years who were treated increased from 0.6% in 2001 to 2.3% in 2011 (Svedbom et al., 2013).

The number of women over 50 with OP in the Czech Republic in 2010 is estimated 426 000. Around 80% of all patients treated for OP used bisphosphonates in 2013 (Fuksa and Vytrisalova, 2015).

The decision to evaluate bone mineral density (BMD) should be based on evaluation of fracture risk as a whole and skeletal health (Cosman et al., 2014). Patients with a prior fracture should be considered for treatment without the need for further risk assessment although BMD measurement may be appropriate, particularly in younger postmenopausal women (Kanis et al., 2013).

In the Czech Republic, many classes of medications are attributed to specific obligatory prescribing conditions (which specialists are entitled to prescribe respective medications with reimbursement, and/or for what subpopulation or disease states, it may be prescribed with reimbursement). Antiresorptive drugs may be prescribed by physicians specialized in rheumatology, endocrinology, orthopedics, clinical osteology, internal medicine, and gynecology for patients with OP (T-score below −2.5 SD) or for patients who experienced low-trauma fracture. Prescribing conditions of most antiresorptives have been liberated to allow for prescribing by general practitioners (GPs), i.e., after initial check-up by a specialist, prescription can be delegated to GP.

Our survey carried out in 2007 (baseline survey) showed insufficient knowledge and engagement in care for patients at risk of fracture with most commonly reported barriers to be financial limits set by health insurance companies and lack of authorization to prescribe antiresorptive drugs (Blazkova et al., 2010, 2012; Vytrisalova et al., 2014). Since then, “delegated prescription” was introduced, the guideline for GPs covering diagnosis and treatment was updated (Palicka et al., 2011), and educational activities with an emphasis on problematic areas were organized. Therefore, we expect improved knowledge of OP.

Otmar et al. interpret their qualitative study among GPs as follows: primary prevention in women younger than 65 years is relegated to a utopian healthcare system—an ideal world in which sufficient time, resources, and motivation are available (Otmar et al., 2012). That's why we focused particularly on secondary prevention in this survey. The aim was to evaluate selected areas of adherence to OP management. We focused on knowledge about OP and its connection with adherence to the guideline, steps taken after a fracture, and potential barriers in management among Czech GPs. Furthermore, to see the development in OP management in primary care, we discussed differences observed in comparison with the baseline survey.

## Methods

**Figure 1:**
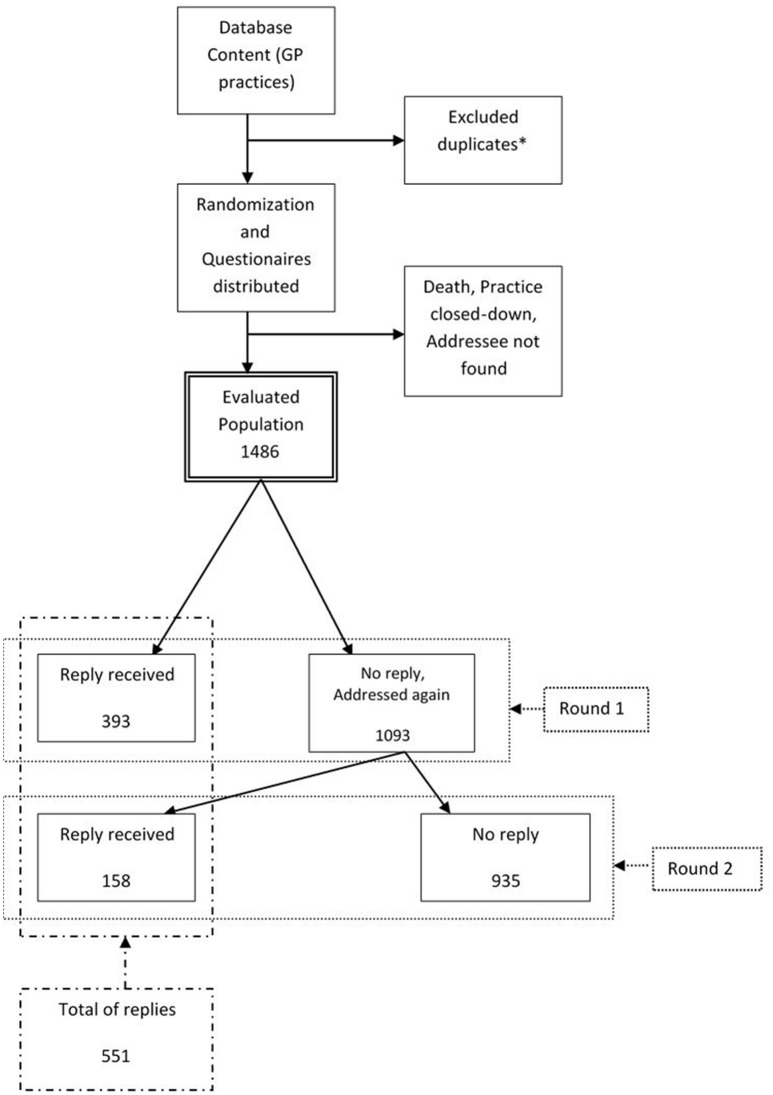
**Response outcomes of the survey**. General practitioners who provide their services at more than one location were contacted at one address only.

On behalf of Society of General Practice and Society for Metabolic Skeletal Diseases, Czech Medical Association JEP, a postal questionnaire survey among Czech GP's was performed. Questionnaires were distributed to GPs randomly selected from a database of the Institute of Health Information and Statistics of the Czech Republic from September to November 2014. Those who failed to reply within 3 weeks (first round) were addressed again (second round). All questionnaires received within 2 months from the start of the second round were included in analyses. The flow of the responses through the study is shown in Figure 1. The study protocol has been approved by Ethics Committee, Faculty of Pharmacy in Hradec Kralove, Charles University.

Questionnaire form consisted of nine multiple choice questions and was sent together with stamped reply envelope. A cover letter signed by heads of the above mentioned professional associations was included. Besides demographic data, i.e., age, gender, length of professional experience, and community size, the questionnaire covered areas concerning (Kanis et al., 2013) the GP's role in the fight against OP, Svedbom et al. (2013) knowledge about OP, Štěpán et al. (2015) management of osteoporosis-related fractures, Stepan et al. (2012) barriers to the management of OP, and Fuksa and Vytrisalova (2015) availability and use of information sources.

Knowledge about OP included risk factors (13 items with yes/no response alternatives) and recommended calcium and vitamin D intake (2 items with open responses). One point was assigned to each correct, minus one point to each incorrect, and 0 to unmarked response. Knowledge score was calculated as a sum of all values assigned to each response. Maximum of the knowledge score was therefore 15 points.

Regarding the management of fractures, GPs were asked about steps which are taken when facing a patient with a fracture as a consequence of OP (multiple choice).

### Statistical analysis

Since most variables did not follow a normal distribution, non-parametric statistics were used. Associations between variables were studied as follows:

1. Simple chi-square test for two dichotomous variables,
2. Kendall correlations for two continuous (ordinal) variables,
3. Mann-Whitney test for dichotomous and continuous (ordinal) variables.

Statistical analyses were calculated using PASW software (version 18.0). P < 0.05 was considered statistically significant.

## Results

The overall questionnaire return rate was 37% (551 respondents); 33% in men and 39% in women, respectively. Characteristics of respondents are summarized in Table 1.

**Table 1:** **Characteristics of the respondents**.

**Age**	***N* = 545**
Mean, median (range)	53, 55 (27–82)
**Length of professional experience**	***N* = 530**
Mean, median (range)	25, 26 (1–57)
**Gender**	***N* = 545**
Men (%)	37
**Community size**	***N* = 541**
<2,000 population (%)	13.3
2,000–10,000 population (%)	32.3
10,000–100,000 population (%)	34.0
>100,000 population (%)	20.3
**Self-perceived GP's role in the fight against OP**	***N* = 546**
Small (%)	5.3
Medium (%)	61.0
Essential (%)	33.7

The GP's role in the fight against OP is rated as essential by 28 and 37% of men and women, respectively (*P* = 0.012). The GP's role was perceived as more important by respondents from larger communities (*P* = 0.039) and was not associated with either age or length of professional experience.

### Adherence to information sources

**Figure 2:**
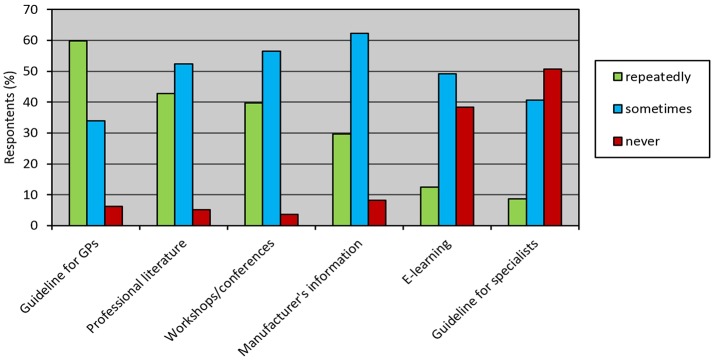
**Use of information sources about osteoporosis among 551 general practitioners in CZ**. Adherence to guideline for GPs was the highest: approximately 60% of respondents reported repeated use.

The guideline for diagnosis and treatment of OP for GPs was considered accessible by 92% of respondents. Use of the guideline compared to other information sources is demonstrated in Figure 2.

### Knowledge

As much as 41 and 40% of respondents stated correctly daily intake of calcium and daily intake of vitamin D, respectively, recommended for postmenopausal women. Most respondents were knowledgeable of risk factors for OP (Table 2).

**Table 2:** **Knowledge of risk factors for osteoporosis or osteoporosis-related fracture, *N* = 551**.

**Risk factor**	**Knowledgeable respondents (%)**	**Incorrect answer (%)**	**Not stated (%)**
Glucocorticoid therapy	97.6	0.0	2.4
Age	94.7	0.4	4.9
Preterm menopause	94.4	0.9	4.7
Immobilization, lack of physical activity	93.1	1.1	5.8
History of low-trauma fracture after age of 45 years	91.8	0.9	7.3
Anorexia nervosa	74.8	4.2	21.1
Smoking	72.6	7.3	20.1
Decreased height	70.6	6.2	23.2
Low body mass index	59.0	12.5	28.5
History of hip fracture in the mother	51.7	14.0	34.3
Type 1 diabetes	27.6	26.0	46.5

The mean knowledge score was 7.7 ± 2.9 (51% of possible points) out of 15 points. The knowledge score correlated negatively with age (*P* = 0.003) and length of professional experience (*P* = 0.011): mean 8.7 points (58%) in the youngest decile vs. 7.0 points (47%) in the oldest decile. The knowledge score was associated with the higher perceived significance of the GP's role in fight against OP (*P* < 0.001) and was not associated with community size. Knowledge of the risk factors, such as anorexia nervosa, smoking, and low body mass index (*p* < 0.01 for each) as well as knowledge score itself (*P* < 0.027) was better in women than in men.

### Steps taken after a fracture

The steps taken by respondents when facing a patient with fracture as a consequence of OP are summarized in Table 3.

**Table 3:** **Actions after a fracture**.

**Option**	**Respondents (%)**
I refer the patient to a specialist, if not yet under follow up^*^	86.2
I recommend lifestyle changes (appropriate diet, physical activity)	76.0
I prescribe calcium/vitamin D supplements	72.5
I provide instruction on fall prevention	49.4

* *Specialist was further specified as follows: osteologist (N = 219), orthopedist (N = 82), rheumatologist (N = 64), DEXA measurement (N = 59), endocrinologist (N = 48), internist (N = 26), and gynecologist (N = 12)*.

*Question: What steps do you take when facing a patient with fracture as a consequence of osteoporosis? (Multiple choice); N = 549*.

### Barriers

Perceived barriers to OP management are listed in Table 4. The most commonly stated ones were lack of possibility to prescribe selected drugs (61%) and financial limits set by health insurance company (44%). Adverse effects of medications were more frequently perceived as a barrier in elderly respondents (*p* = 0.010). On the contrary, other health problems perceptive as more serious (*p* = 0.011) and lack of time (*p* = 0.040) were more frequently marked by younger respondents.

**Table 4:** **Barriers in the fight against osteoporosis**.

**Barrier**	**Respondents (%)**
GP is not authorized to prescribe selected drugs	60.7
Financial limits set by the health insurance company	43.9
Patient's non-adherence	28.7
Prioritized health problems (perceptive as more serious)	27.8
OP should be managed by a specialist	25.0
Unavailability of diagnostic examinations	18.7
Communication with specialists, delayed or no reports	17.8
Patient's reluctance to go to a specialist	17.6
Lack of time	17.2
Inadequate knowledge of OP	16.5
Unavailability of specialized treatment	13.0
Adverse effects of medications	9.8

*Question: The main limitations to your fight with osteoporosis are (multiple choice), N = 540*.

## Discussion

This survey among GPs is a simplified follow-up to our previous baseline survey with similar goals and organization (Blazkova et al., 2010, 2012; Vytrisalova et al., 2014). So far, certain attention to the topic of OP management among GPs has been payed (Taylor et al., 2001; Pérez-Edo et al., 2004; Chenot et al., 2007; Cortet, 2009; Dorner et al., 2009; Bruyère et al., 2013), however, fresh longitudinal studies monitoring development in this issue have been lacking. Similarly to 2009 Austrian survey relating to a preceding 1993 survey (Dorner et al., 2009), here we also report a marked improvement in a number of evaluated items compared to the previous 2007 situation.

Response rate of 37%, similar to previous 2007 survey (38%), was relatively high, compared to recent surveys among GPs in which the rates were up to one third of addressees. In Belgium, the response rate of 28% in the survey on perception and knowledge of the FRAX tool (Bruyère et al., 2013) and 30% in the survey on vitamin D supplementation prescription in nursing homes (Buckinx et al., 2016) was achieved. A national survey regarding sun exposure and vitamin D in New Zealand, which included more than one thousand GPs, achieved 32% (Reeder et al., 2012).

Perception of GPs' role in the fight against OP and its associated factors were also similar to our previous survey (Blazkova et al., 2010). Around 35% of respondents in both surveys considered their role as essential. Women and respondents from larger communities rated it as significantly more important than men and respondents from smaller communities. This could be explained by health education including those related to OP organized particularly in urban areas. Furthermore, female gender and more comfortable life style poor in physical activity are risk factors.

Similar to the baseline survey, the dominant source of information was guideline for GPs issued by Society of General Practice, Czech Medical Association JEP. This corresponds with efforts of the society which regularly sends all new guidelines as short well-designed booklets by mail to all GPs, and the guidelines are also freely available on the website of the society. The guideline was used repeatedly by 60% of respondents, which is slightly more than in 2007 (Table 5), and more than 19 of respondents found it easily accessible. The guideline use was followed by professional literature and conferences, both were used repeatedly by around 40% of respondents. Compared to the baseline survey, slightly more respondents also used guideline for specialists issued by Society for Metabolic Skeletal Diseases. Increased frequency of use of guidelines was accompanied by better knowledge and higher initiative in post-fracture care reported in the present survey, which is a positive trend.

**Table 5:** **Comparison of the findings of the present survey (2014) with our baseline (2007) survey (Blazkova et al., 2010, 2012; Vytrisalova et al., 2014)**.

**Item of the survey**	**2007 (*N* = 525)**	**2014 (*N* = 551)**
**INFORMATION SOURCES**		
Guideline for GPs used repeatedly (%)	54	60
**KNOWLEDGE OF RISK FACTORS**		
Smoking (%)	55	73
Low body mass index (%)	40	59
Anorexia nervosa (%)	56	75
History of hip fracture in the mother (%)	45	52
**KNOWLEDGE OF RECOMMENDED INTAKE**		
Calcium (%)	11	41
Vitamin D (%)	Not included	40
**SECONDARY PREVENTION–STEPS AFTER A FRACTURE**		
Instruction on fall prevention (%)	39	49
Prescribing of calcium/vitamin D (%)	61	73
Recommendation of life-style changes (%)	64	76
**BARRIERS (SYSTEM-RELATED)**		
GP not authorized to prescribe selected drugs (%)	71	61
Financial limits set by the health insurance company (%)	71	44
**BARRIERS (PATIENT-RELATED)**		
Patient's non-adherence (%)	16	29
Patient's reluctance to go to a specialist (%)	8	18
Other health problems (more serious and prioritized) (%)	15	28
**BARRIERS (PHYSICIAN-RELATED)**		
Lack of knowledge (%)	11	17
OP should be managed by a specialist (%)	20	25

*Only items with 5% or higher difference (rounded to the nearest whole percent) are presented*.

We found out, in accordance with baseline survey again, very good knowledge, i.e., more than 90% of knowledgeable respondents, concerning main risk factors, such as age, glucocorticoid therapy, immobilization, and preterm menopause. Strong awareness of glucocorticoid therapy as a risk factor was observed also in qualitative study among GPs in Australia (Otmar et al., 2012) and in the study from the Netherlands (Duyvendak et al., 2011). Furthermore, respondents, women in particular, were more aware of risk factors which were roughly underrated in 2007 (Table 5). These include smoking, low body mass index, and anorexia nervosa. Improvement in knowledge of each factor by 15% is encouraging but still insufficient. Family history appears to be constantly undervalued. Better awareness of risk factors could be helped by wider use of FRAX, a simple tool that calculates fracture probability (available from https://www.shef.ac.uk/FRAX). It is based on individual risk factors and country-specific fracture epidemiology. Although mentioned in the guideline for GPs, Czech reimbursement rules and prescribing conditions do not take into account FRAX-based evaluations, and its use is rare in the Czech Republic even in comparison with Poland, Hungary or Slovakia (Kanis et al., 2014).

As many as 40% of respondents were knowledgeable of total daily intake of calcium and vitamin D recommended for postmenopausal women. This is also much better compared to the baseline survey, nevertheless still insufficient (Table 5). One reason could be that much inconsistency exists regarding the optimal doses and forms of calcium and vitamin D. In the survey among GPs from New Zealand, which was focused on sun exposure and vitamin D, 43% of respondents were not at all confident about vitamin D knowledge (Reeder et al., 2012).

Improvement probably reflects impact of educational activities regarding OP organized for GPs in recent years, increasing adherence to the guidelines, and increasing attention devoted to the topic in medical literature. As mentioned above, it is possible that women are more interested in OP since they perceived it a condition typically affecting them. Therefore, women can be more motivated to education or (at least) to find respective information at the time of the survey and mark correct answers due to social desirability. Gender-specific perception of OP-related topics has been published: e.g., greater concern about vitamin D intake than about skin cancer was expressed by women in the above mentioned survey (Reeder et al., 2012). Knowledge of several risk factors better in women corresponds with higher response rate and perception of the role in management of OP in women than in men. The gender difference was more remarkable than in the baseline survey. Taking into account rapid growth of medical knowledge and increasing demands on primary care, tendency of GPs to be more aware of conditions of personal interest is understandable. Despite much better knowledge compared to the baseline survey, lack of knowledge was perceived as a barrier by more respondents than in 2007 (Table 5).

Although younger GPs had better knowledge, similarly to other surveys including our baseline (Pérez-Edo et al., 2004; Blazkova et al., 2010), this merit (also similarly to the baseline; Blazkova et al., 2010) was not accompanied by higher self-reported quality of care i.e., activities when facing a patient with a fracture. Neither of active steps after a fracture was associated with age.

Respondents actively cared for patients after a fracture. Compared to the baseline survey, there was an obvious increase (by 10% of respondents) in performing all key steps, such as giving instructions on fall prevention, prescribing calcium/vitamin D and recommendation of lifestyle changes (Table 5). Slightly more GPs also refer a patient after a fracture to a specialist, if not yet under follow-up. This can be also associated with trust in DXA measurement as found in the study of Otmar et al. in which GPs expressed a high level of confidence in DXA, both as diagnostic and monitoring tool (Otmar et al., 2012).

Almost three quarters of respondents stated they prescribe calcium/vitamin D supplements to their patients after a fracture. In the survey of Buckinx, more than half of GPs systematically prescribe vitamin D to their patients in nursing homes (Buckinx et al., 2016). However, 60% of the respondents is not aware of the recommended dosing of the nutrients. It is not known whether the prescribing is appropriate (sufficient) with respect to dietary intake of a particular patient. Appropriate prescribing of the supplements is therefore probably still far from optimum. Uncertainty regarding adequate dosing could be caused by rapidly increasing clinical evidence of higher doses of vitamin D (≥800 IU a day) than used in the past, low dose of vitamin D in most combined supplements available at the time of the survey, and concerns about cardiovascular risk of calcium (Kanis et al., 2013).

As in the baseline survey, the most frequently stated barriers were lack of possibility to prescribe selected drugs and financial limits set by health insurance companies. However, perception of these regulations as barriers, compared to the baseline survey, decreased by 14 and 38%, respectively (Table 5). Reasons can be liberation of prescribing of antiresorptives and less strict budget limits. In the Czech Republic, prescribing conditions (for drugs to be reimbursed) play a decisive role in treatment algorithms. The reason is low willingness of patients to pay for drugs out-of-pocket and no private health insurance. In 2007, no antiresorptive and osteoanabolic drugs could be prescribed by GPs i.e., they could be prescribed exclusively by physicians with selected specializations. Until 2014, these conditions were liberated considerably, so in 2014 GPs could prescribe bisphosphonates and some other less utilized antiresorptives, such as stroncium ranelate, and raloxifene (Fuksa and Vytrisalova, 2015). Even though, the therapy must be initially indicated by a specialist. Naturally, this does not necessarily imply that prescription rate by GPs rose considerably and/or that GPs started using the new options widely, but the potential barrier and its perception might, at least partially, have been lifted.

Annual prescription budget limits placed upon the physicians by the insurance companies are often perceived as a barrier (Chenot et al., 2007). This “limit” essentially means that any single GP's spending (on prescription drugs separately) is benchmarked within a period (year) with all other GPs, as well as his/her spending is benchmarked with the same GP's spending in different years. Then those physicians spending overly (often due to prescribing expensive drugs) are penalized. These mechanisms are complex and regulated by many factors, starting from Czech Ministry of Health's annual decrees, which reflect the economic situation of the health care insurance budgets, and ending in the individual financial contracts between physicians and health insurance companies. Comparing the abovementioned annual decrees issued in 2007 and 2014 it is apparent that both the benchmarking rules and the limits changed: they were less strict in 2014. Also many of the concerned drugs, bisphosphonates in particular, saw a massive influx of generics followed by reimbursement price revisions leading to price decline between 2007 and 2014. For instance, reimbursement price of ibandronate (and other bisphosphonates) decreased by 60% in that period. Both factors point to the fact, that the GPs possibly perceived the threat of “breaking the prescription budget” as less important in 2014 than they did in 2007 (Table 5).

As mentioned above, treatment of OP can be provided by a GP in cooperation with a specialist, who can delegate prescription of selected antiresorptive drugs to the GP. Communication between GPs and specialists is probably relatively well managed; less than one fifth of the respondents (and also in the baseline survey; Blazkova et al., 2010) considered it as a barrier. However, one quarter of respondents believe OP should be managed by a specialist, and more than one quarter reported that more serious health problems are a barrier. These numbers are remarkably higher than in the baseline survey (Table 5) which corresponds with referrals to a specialist after a fracture reported also slightly more frequently than in 2007. Otmar et al. (2012) also found out that OP was something the GPs were not particularly concerned about, regarding their patients or the population as a whole. They ranked their own concerns about adults living with diabetes, osteoarthritis, cardiovascular disease, hypertension, cancers and depression higher than concern about OP.

Patient-related barriers almost doubled compared to our baseline survey (Table 5). One reason could be cost of therapy, even though relatively low. Most preparations containing calcium, have switched to over-the-counter (OTC) drugs in the Czech Republic, however there are still fixed combinations almost fully covered. It is also possible patients have problems with unusual medication regimens with prolonged dosing intervals. Most common antiresorptive drugs are oral bisphosphonates which should be used in a special way to ensure adequate absorption and avoid gastrointestinal irritation, separately from calcium supplements. Implementation of such regimens into daily routine, particularly in elderly with multiple therapy, can be of concern. Taking into account the asymptomatic nature of the disease, respondents stated that there are conditions perceived as more serious. Therefore, patients at risk of fracture, if not properly informed and motivated (e.g., using results of DXA monitoring) need not be adherent enough and/or willing to visit a specialist.

A random sample of GPs (more than one quarter of all Czech GPs) was addressed on behalf of the two most relevant professional associations, which was probably the reason of relatively high response rate. The rate, however, does not ensure a representative sample of Czech GPs. Factors which should be considered when extrapolating to all GPs are, similarly to other surveys, probable bias of volunteering (less knowledgeable GPs did not participate in the survey) and social desirability bias (tendency to provide answers which are expected by the investigator). However, potential overestimation of the knowledge and activities is probably very similar to the baseline survey, since the same method was used and similar response rate was achieved. The results provoke hypotheses possibly calling for further investigation, such as tracking real-world evolution of prescribing rate of antiresorptive drugs by GPs, which would help to uncover the changes in the barrier of prescribing. Such data analyses were not in scope of the present survey therefore may be a topic for future research.

## Conclusion

GPs adhered to the selected areas of OP management more than in 2007 (baseline survey). Knowledge of most risk factors for OP was relatively high and much better than in 2007, probably due to educational activities targeted at GPs and the updated guideline. Relatively high self-estimated adherence to secondary prevention was observed. GPs reported to be more involved in post-fracture care, i.e., recommendation of lifestyle changes, prevention of falls, referral to a specialist, and prescribing calcium/vitamin D supplements. However, awareness regarding intake of calcium and vitamin D, and several risk factors (family history in particular) was still insufficient. Systemic regulations, such as liberation of prescribing conditions, less strict annual prescription budget limits, and influx of generics of bisphosphonates probably helped much to eliminate major obstacles in the management of OP perceived by GPs. However, impact of delegated prescription and prescribing of antiresorptives by GPs are unknown. Prescribing conditions therefore remain an important issue. Compared to the baseline survey, patient-related barriers to the management, patient non-adherence in particular, were more common, which seems striking. Education focused on calcium and vitamin D intake (doses, sources, and supplements), and support of the FRAX instrument use would be appropriate. It seems important to increase awareness about OP and its consequences among general public, elderly in particular, and to study patient-related barriers in detail.

## Author contributions

MV: Conceptualized and organized the study. Carried out literature review, handled data, performed statistics, interpreted the results, drafted and revised the manuscript. TT: Handled the patient data and prepared the data for analysis. Carried out analyses and statistics. Prepared the figures. Drafted and revised the manuscript. LF: Conceptualized the study. Interpreted the results, revised the manuscript. RK: Organized the study. Handled the patient data and prepared the data for analysis. Carried out analyses and statistics. Prepared the figures and revised the manuscript. VP: Conceptualized the study. Contributed to interpretation of the results and revised the draft. SB: Conceptualized the study. Contributed to interpretation of the results and revised the draft. JS: Conceptualized the study. Contributed to interpretation of the results and revised the draft.

## Funding

The study was supported by Charles University in Prague, the projects SVV 260 295, SVV 260 417, by MH CZ–DRO (UHHK, 00179906) and by the programme PRVOUK P37/11.

### Conflict of interest statement

The authors declare that the research was conducted in the absence of any commercial or financial relationships that could be construed as a potential conflict of interest.
